# Fungal Microbiota of Sea Buckthorn Berries at Two Ripening Stages and Volatile Profiling of Potential Biocontrol Yeasts

**DOI:** 10.3390/microorganisms8030456

**Published:** 2020-03-23

**Authors:** Juliana Lukša, Iglė Vepštaitė-Monstavičė, Violeta Apšegaitė, Laima Blažytė-Čereškienė, Ramunė Stanevičienė, Živilė Strazdaitė-Žielienė, Bazilė Ravoitytė, Dominykas Aleknavičius, Vincas Būda, Raimondas Mozūraitis, Elena Servienė

**Affiliations:** 1 Laboratory of Genetics, Institute of Botany, Nature Research Centre, Akademijos str. 2, LT-08412 Vilnius, Lithuaniaigle.vepstaite@yahoo.com (I.V.-M.); ramune.staneviciene@gamtc.lt (R.S.); zivile.strazdaite-zieliene@gamtc.lt (Ž.S.-Ž.); bazile.ravoityte@gamtc.lt (B.R.); 2 Laboratory of Chemical and Behavioral Ecology, Institute of Ecology, Nature Research Centre, Akademijos str. 2, LT-08412 Vilnius, Lithuania; violeta.apsegaite@gamtc.lt (V.A.); laima.blazyte@gamtc.lt (L.B.-Č.); dominykas.aleknavicius@gamtc.lt (D.A.); vincas.buda@gamtc.lt (V.B.); raimondas.mozuraitis@gamtc.lt (R.M.)

**Keywords:** *Hippophae rhamnoides*, fungal communities, volatile organic compounds

## Abstract

Sea buckthorn, *Hippophae rhamnoides L*., has considerable potential for landscape reclamation, food, medicinal, and cosmetics industries. In this study, we analyzed fungal microorganism populations associated with carposphere of sea buckthorn harvested in Lithuania. An amplicon metagenomic approach based on the ITS2 region of fungal rDNA was used to reveal the ripening-affected fungal community alterations on sea buckthorn berries. According to alpha and beta diversity analyses, depending on the ripening stage, sea buckthorn displayed significantly different fungal communities. Unripe berries were shown to be prevalent by *Aureobasidium*, *Taphrina*, and *Cladosporium*, while ripe berries were dominated by *Aureobasidium* and *Metschnikowia*. The selected yeast strains from unripe and mature berries were applied for volatile organic compounds identification by gas chromatography and mass spectrometry techniques. It was demonstrated that the patterns of volatiles of four yeast species tested were distinct from each other. The current study for the first time revealed the alterations of fungal microorganism communities colonizing the surface of sea buckthorn berries at different ripening stages. The novel information on specific volatile profiles of cultivable sea buckthorn-associated yeasts with a potential role in biocontrol is important for the development of the strategies for plant cultivation and disease management, as well as for the improvement of the quality and preservation of the postharvest berries. Management of the fungal microorganisms present on the surface of berries might be a powerful instrument for control of phytopathogenic and potentially antagonistic microorganisms affecting development and quality of the berries.

## 1. Introduction

Sea buckthorn (*Hippophae rhamnoides L*.) is garden and landscaping shrub widespread in Europe, Asia, and North America, distinguished for good adaptation to barren soils and harsh climates [1]. The ecological impact of sea buckthorn in preventing soil erosion, improving its properties, and reducing pollution has been demonstrated [2]. Economically, the plant species has been considered as a source of healthy nutritious food, giving rise to an increased demand for medicine and cosmetics [3,4]. Cultivation of sea buckthorn is developing due to the request for its berries with a high content of minerals, different vitamins, specific valuable oils, phenolics, and essential fatty acids, as well as low level of both fructose and glucose [5]. Phytochemicals and essential oil generated from sea buckthorn berries and seeds reduce inflammation; have antibacterial, anticancer, and radioprotective activity; relieve pain; and promote regeneration of tissues [3,6,7]. 

The microenvironments coherent with plants and fruits are highly colonized by different communities of bacterial and fungal microorganisms [8]. Plants provide nutrient-rich and stable surrounding conditions vital for the development of microbiota. In turn, epiphytic microorganisms produce secondary metabolites that may enhance plant immunity to unfavorable conditions and diseases [8,9,10]. Numerous microorganisms due to the production of volatile organic compounds (VOCs) have been found to promote plant growth and improve crop productivity [11]. Volatile production, secretion of antifungal compounds, and killer toxins allow for fruit-associated microorganisms to demonstrate increased antagonistic activity, an ability to modulate carposphere microbiota structure, and might mediate interactions with insects [12,13,14,15,16]. The role of bacterial and fungal microorganisms as producers of chemical indicators for the attraction of insects and their communication has also been revealed [17,18]. Microorganisms occupy a trophic level between plants and insects. Insects from several orders are feeding on fruits, while yeasts use insects either as hosts or vectors [17]. The chemical composition of each blend of volatiles may vary depending on the producing yeast and the ecological niche where the cross-talking species are growing [14,16].

The spreading of microorganisms on plants is determined by many factors, such as climatic conditions, application of agrochemicals, plant species, and ripening stage [19,20,21]. Changes in the planting regime alter microbial habitat and diversity, affect functional traits [22]. Comprehensive next-generation sequencing-based (NGS) microbiome analysis accomplished on several fruits and berries—such as apples, blackcurrants, grapes, olives, strawberries, oranges, etc.—is mainly focused on plant-conditioned prevalence [23,24,25,26,27,28]. The biogeographic distribution of microbiota communities has been revealed on apple, blackcurrant, and grape [21,28]. The effect of the ripening stage on the development of the microbial population was investigated in grapes, mango, nectarines and plums [8,29,30,31]. Analysis of grape bacterial microbiota defined changes in the structure and size of the population occurring during the ripening process. The levels of bacteria raised gradually and reached their highest value when the berries became overripe [8]. Yeasts are sparsely observed in the early stage of fruit development, but they increase in number as fruits ripen [32]. The yeast genera occurring on most mature nectarine fruit (e.g. *Hanseniaspora*, *Pichia*, and *Zygosacharomyces*) were significantly different from those on fruit at earlier stages of development [29]. 

It must be noticed that few works dealing with the characterization of sea buckthorn-associated microbial communities were published [33,34]. By analyzing representative microbial operational taxonomic units (OTUs) prevalent on sea buckthorn berries, the plant-defined composition of bacterial and fungal microbiota was recorded [33]. The impact of plant age and season was investigated in sea buckthorn plantation on the rhizosphere microbial community only [34,35]. 

To the best of our knowledge, there is no information on the ripening affected sea buckthorn carposphere-associated fungal community’s alterations and release of volatile organic compounds by the yeasts isolated from this plant. Therefore, the aims of this study were (i) to investigate the changes of the sea buckthorn-associated fungal microbiota depending on the ripening stage of the berries, (ii) to isolate and cultivate potential biocontrol yeasts distributed on unripe and ripe sea buckthorn berries, and (iii) to identify pattern of volatiles produced by the selected cultivable yeast species. The obtained information on the ripening-affected changes of sea buckthorn-associated fungal microbiota composition, and distribution of potentially beneficial and phytopathogenic microorganisms, may be important to develop effective plant disease control strategies. Due to high demand of sea buckthorn berries in food industry and increased request of unprocessed food, the metagenomics data appear to be highly relevant for the evaluation of the impact of microbiota and specific microorganisms on food quality and human health. Isolated cultivable yeasts might encourage new opportunities for developing natural tools for disease management, postharvest protection, and the quality of the sea buckthorn berries with potential in food production. The data on volatile profiling of selected yeasts could be relevant for improving plant productivity and generating new environmentally friendly formulations for pest control and plant disease management. 

## 2. Materials and Methods

### 2.1. Ethics Statement

The collection of sea buckthorn berries was authorized by the private owner, fully acknowledged in a paper. Endangered or protected species were not involved; therefore, no specific permissions were required.

### 2.2. Sample Collection

*Hippophae rhamnoides L.* berries were aseptically sampled from the organic private plantation located in the Molėtai region of Lithuania (GPS coordinates: 55°15′12.2”N, 25°26′23.1″E). The sea buckthorn berries were collected from three different places of sea buckthorn plantation separated by more than 50 meters. The berries were randomly selected from three bushes and combined into one biological replicate at each location. The experiment was carried out at two different growth stages: at the beginning of berry ripening (UB) in July and ripe berries (RB) in September 2018. The samples were collected into sterile plastic bags, transported to the laboratory and processed within 2–3 h after harvesting. 

For metagenomic analysis, 300 g of the berries were placed into flasks with 500 mL of sterile 0.05M phosphate buffer (pH 6.8) for 30 min and incubated at room temperature with shaking at 120 rpm. Outwashes were filtered through 420 μm filters and followed centrifugation at 12,000× g for 20 min. The pellets were stored at −20 °C for subsequent DNA extraction. 

For cultivable fungal microorganisms isolation, 15 g of the berries were placed in a 30 mL of liquid MD medium (2% dextrose, 1% (NH4)2SO4, 0.09% KH2PO4, 0.05% MgSO4, 0.023% K2HPO4, 0.01% NaCl, 0.01% CaCl2) for 24 h at room temperature with shaking at 100 rpm. Outwashes were serially diluted in MD medium and plated on extract-peptone-dextrose (YPD)-agar plates (1% yeast extract, 1% peptone, 2% dextrose, 2% agar) containing 50μg mL^−1^ chloramphenicol and incubated for 3–5 days at 25 °C. Morphologically distinct yeast-like colonies were applied for molecular identification.

### 2.3. Metagenomic Analysis of Sea Buckthorn Fungal Microbiota

For metagenomic analysis, DNA was isolated from collected sediments (40 mg of pellet per sample) using a Genomic DNA purification kit (Thermo Fisher Scientific Baltics, Vilnius, Lithuania) and following the manufacturer’s protocol. The quantity and quality of extracted DNA were measured using a Nanodrop 2000 spectrophotometer (Thermo Fisher Scientific). DNA samples of fungal microorganism populations were amplified using the ITS2 region-specific primers: ITS3-KYO2 (5′-GATGAAGAACGYAGYRAA-3′) and ITS4 (5′-TCCTCCGCTTATTGATATGC-3′) [36]. Amplicon libraries were prepared using modified Illumina adapters (www.illumina.com), validated on an Agilent Technologies Bioanalyzer DNA 1000 and paired-end sequenced (2 × 300 bp) using Illumina MiSeq platform (BaseClear B.V. Leiden Netherlands). Complete data sets were submitted to the National Center for Biotechnology Information (NCBI) Sequence Read Archive (SRA) database (Accession number PRJNA590349).

### 2.4. Bioinformatics Analysis

Sequences were pre-processed, quality filtered, and analyzed using QIIME2 version 2018.4 [37]. Forward and reverse reads were trimmed with Cutadapt 2.8 [38]. Primer-free fastq files were processed using Divisive Amplicon Denoising Algorithm 2 (DADA2) plugin implemented in QIIME2. Reads with higher than 2.6 expected error rates were discarded. Chimeric sequences were eliminated using ‘consensus’ method. Alpha and beta-diversity analyses were performed with the q2-diversity plugin in QIIME2 at a sampling depth of 43458. Principal coordinates analysis (PCoA) was performed based on non-phylogenetic Bray–Curtis distances and visualized with the make_2d_plots.py script. To assign taxonomy of obtained Amplicon sequence variants (ASV) we used the QIIME2 q2-feature-classifier plugin and the v7.2 UNITE database as the reference at 97% identity [39]. 

### 2.5. Cultivable Yeast Identification

Yeast strains were streak-plated from single colonies onto YPD-agar medium supplemented with 50 μg mL^-1^ chloramphenicol and incubated for 3 days at 25 °C. The colors and the structure of the colonies were recorded. For the microscopy study, slides were prepared from 1-week-old cultures and observed under the light microscope (Leica DM750, Wetzlar, Germany), by recording micrographs with the digital camera (Leica ICC50 HD, Wetzlar, Germany). 

DNA was isolated from fresh yeast culture (24 h) by using the Genomic DNA purification kit (Thermo Fisher Scientific Baltics, Vilnius, Lithuania) following the manufacturer’s instructions. For identification of yeast, PCR amplification of the region between the 18S rRNA and 28S rRNA genes was performed using ITS1 (5′-TCCGTAGGTGAACCTGCGG-3′) and ITS4 (5′-TCCTCCGCTTATTGATATGC-3′) primers. The reaction mixtures (50 μL) contained 5 μL of DreamTaq green buffer, 1 μL of 2 mM dNTP mix, 1 μL of each primer (10 μmol L^-1^), 2.5 unit of DreamTaq DNA polymerase (all from Thermo Fisher Scientific Baltics, Vilnius, Lithuania) and 1 μL of DNA template (5 ng). The PCR conditions were as follows: initial denaturation at 94 °C for 5 min; 25 cycles of 94 °C for 1 min, 53 °C for 1 min 30 s, 72 °C for 2 min; a final elongation at 72 °C for 10 min. For preliminary yeast species identification, PCR products were digested with *Cfo*I and *Hinf*I enzymes and restriction profiles were checked by 1% agarose gel electrophoresis. PCR products were purified using a GeneJet PCR purification kit (Thermo Fisher Scientific Baltics, Vilnius, Lithuania), following the manufacturer’s instructions and sequenced using ITS1 and/or ITS4 primers at BaseClear (Leiden, Netherlands). The generated sequences were compared with those found in the FASTA network service of the EMBL-EBI database (http://www.ebi.ac.uk/Tools/sss/fasta/nucleotide.html) and deposited in the National Center for Biotechnology Information (NCBI) under accession numbers MN700626, MN700627, MN700628, and MN700629.

### 2.6. Sampling and Identification of Volatile Organic Compounds (VOCs) Produced by Yeasts

The solid-phase micro-extraction (SPME) technique was applied to collect the headspace volatiles of yeasts. Yeasts were cultivated in polystyrene Petri dishes (Ø 55 mm × 14 mm) on YPD-agar (14 mL) for 2 days at 25 °C. For sampling background volatiles, YPD-agar plates without yeast were used as control samples. SPME fiber coated with a polydimethylsiloxane-divinylbenzene polymer (DVB/PDMS, 65 mm coating layer thickness, Supelco, Pennsylvania, USA) was used. Before each collection, purification of SPME fibers was conducted at 240 °C for about 10 min in a GC injector. The needle of SPME syringe was placed above the yeast layer through a small hole made in a Petri dish; the fiber was exposed to the headspace for 60 min at room temperature. After sampling, the fiber was inserted for 2 min into the injection port of gas chromatograph for desorption of the volatiles. 

The volatiles collected were analyzed using Shimadzu gas chromatograph GC-2010 coupled with Shimadzu mass selective detector MS-QP 2010 Plus (Kyoto, Japan). In the GC, Restek Stabil-Wax column (30 m × 0.25 mm × 0.25 μm, Bellefonte, PA, USA) was used. The oven temperature was programmed as follows: the initial temperature was maintained isothermally at 40 °C for 1 min; afterward, it was raised to 200 °C at a rate of 5 °C min-1, then increased to 240 °C at a rate of 10 °C min-1, and maintained isothermally for 11 min. The GC injector temperature was set at 240 °C. Helium was used as carrier gas (1.5 mL min^-1^). The relative amount of each of the compounds was counted based on the area of the chromatographic peak. The volatile compounds were identified according to their mass spectra and their retention indexes in a NIST version 2.0 mass spectral library (National Institute of Standards and Technology, USA) as well as co-chromatography of synthetic standards. Software GCMS solution version 2.71 (Shimadzu, Kyoto, Japan) was applied for data analysis.

### 2.7. Statistical Data Analysis

Nonparametric Mann–Whitney U test was applied to evaluate differences of volatile amounts between yeast and control samples, i.e., YPD-agar plates without yeast, using the Statistica 6.0 program package (StatSoft, Inc., Tulsa, OK, USA). To assess and visualize the associations between odor blends of four yeast species and volatile compounds, the principal component analysis (PCA) was performed using Canoco 4.5 software (Biometris Plant Research International, Wageningen, The Netherlands). Amounts of styrene, 2,5-dimethyl pyrazine, 2-ethylhexanol and methoxy-phenyl-oxime derived from yeast samples did not differ from those collected from the control; hence, they were not included in PCA analysis. Significantly smaller amounts of the compounds collected from the control samples were subtracted from the amounts released by yeasts. By Canoco 4.5 software, absolute amounts expressed as area under chromatographic peak were log-transformed, scaled dividing each value by its standard deviation and centered.

## 3. Results

### 3.1. Diversity and Richness of Fungal Microorganism Communities

To characterize the diversity of fungal microorganism community distributed on sea buckthorn at different ripening stages, we harvested berries from the three distinct shrubs at private organic plantation and generated a combined sample as one biological replicate. For metagenomics analysis, three biological replicate samples of unripe berries (UB) obtained from different locations of the sea buckthorn plantation and three corresponding to ripe berries (RB) were used. Sequencing of the ITS2 rRNA partial gene region of DNA samples generated a total of 726,634 reads with an average of 121,105 sequences per sample (range 897,20-164,922) (Table 1). Following quality filtering, 454,538 sequences were recovered. The clustering of the sequences generated a total of 1615 unique amplicon sequence variants (ASVs) (1014 [338±70, hereafter median for 3 samples ± standard deviation] for fungal ITS2 from unripe berries and 601 [200±35.6] ASV for ripe berries). The observed number of ASV reveals a significant difference in fungal community richness between UB and RB sample groups (ANOVA p=0.038). 

**Figure 1:**
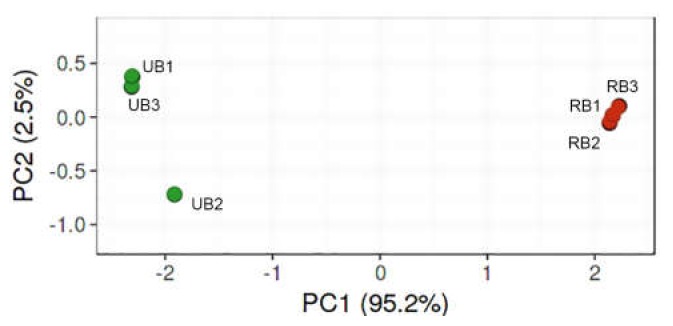
Principal coordinate analysis (PCoA) of the relative abundance of fungal microorganism amplicon sequence variants (ASVs) associated with unripe (UB) and ripe (RB) sea buckthorn berries. PCoA plot is based on unweighted UniFrac distance metrics.

The microbial community analysis according to the nonparametric Kruskal–Wallis test showed a statistically significant decrease (*p*-value 0.046) in fungal microorganism diversity amongst RB samples in comparison to UB samples. In agreement with ASV data, Shannon’s diversity and Simpson indexes estimate that unripe sea buckthorn berries have a higher fungal microorganism diversity than ripe berries. Rarefaction curve approached plateaus indicating that the sequencing depth was sufficient to capture the microbial diversity of each sample (Figure S1). Principal coordinate analysis (PCoA) accomplished with the representative ASVs demonstrated a clear separation of UB and RB samples, indicating a difference in the composition of the fungal microbiota (Figure 1). Clustering of triplicate sample sequence libraries using the Bray–Curtis dissimilarity index revealed consistency of microbial community structure among replicates.

### 3.2. Composition of Fungal Microbiota on Unripe and Ripe Sea Buckthorn Berries

**Figure 2:**
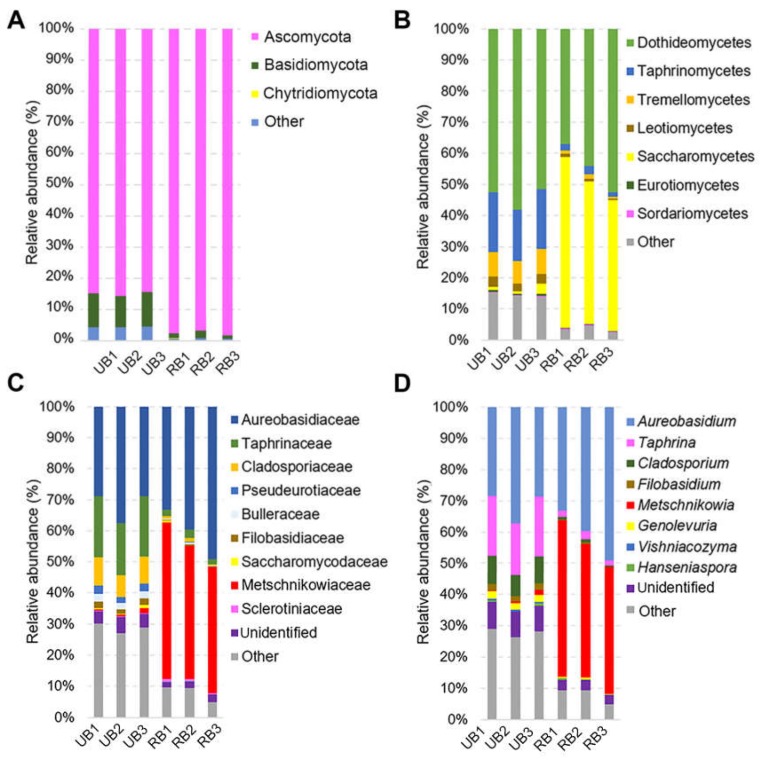
Fungal microorganism community distribution on sea buckthorn unripe (UB) and ripe (RB) berries. Relative abundance of sequences classified at the phylum (**A**), class (**B**), family (**C**), and genus (**D**) level. The taxonomic groups comprising less than 1% of the total composition were assigned to “Other”.

A total of four phyla, 69 orders, and 144 families were identified in the present study (Table S1). Based on the average abundance analysis, the dominating phyla on both sea buckthorn samples were Ascomycota (85.0% for UB and 97.6% for RB, respectively) followed by the Basidiomycota (10.6% and 1.7% in UB and RB, respectively) and other fungi (Figure 2A; Table S1). The impact of ripening on the fungal microorganism community was notable starting at the class level. Dothideomycetes were present in large numbers in both samples (54.0% for UB and 44.6% for RB, respectively) (Figure 2B). However, on unripe berries (UB) the most abundant classes were Taphrinomycetes (18.4%) and Tremellomycetes (7.6%) when in RB samples Saccharomycetes (47.6%) prevailed. At the family level, in UB samples we found dominating Aureobasidiaceae and Taphrinaceae, while RB samples were prevalent by Aureobasidiaceae and Metschnikowiaceae (Figure 2C). In total, 196 genera were identified in this study; the most abundant were *Aureobasidium* (31.4%), *Taphrina* (18.4%), and *Cladosporium* (8.1%) for UB samples, and *Metschnikowia* (44.5%) with *Aureobasidium* (40.6%) for RB samples (Figure 2D). 

**Figure 3:**
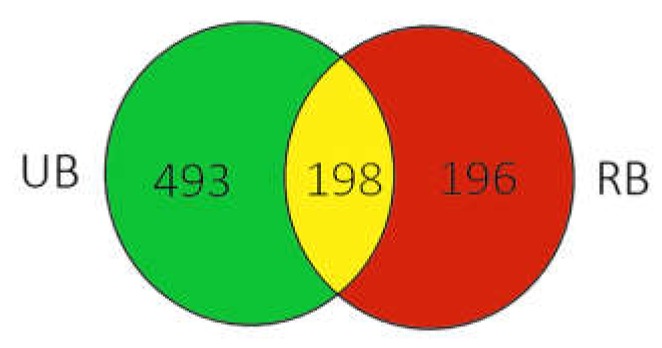
Venn diagram illustrating the number of unique and shared amplicon sequence variants (ASVs) among sea buckthorn samples at different ripening stages. UB – unripe sea buckthorn berries, RB – ripe berries.

The Venn diagram presents the distribution of unique fungal ASVs in UB and RB samples (Figure 3). A total of 887 unique ASVs found in this study, 198 were shared by both UB and RB samples, while 493 ASVs were exclusive to UB and 196 to RB samples, respectively.

**Figure 4:**
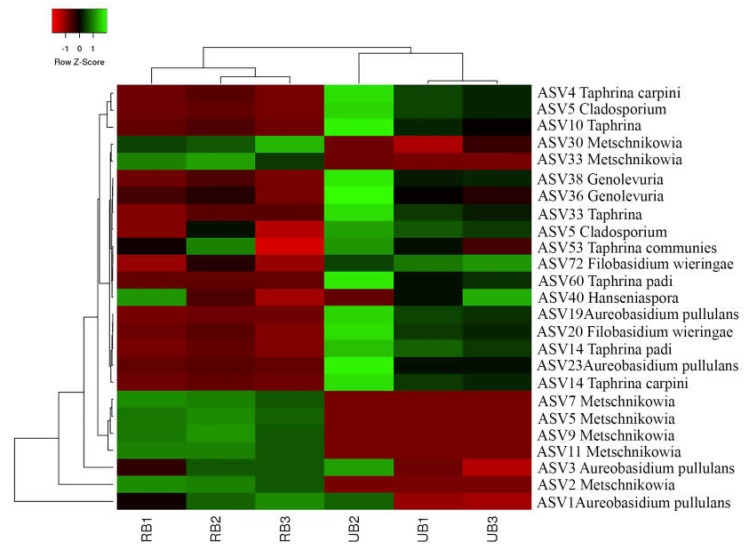
Heatmap of fungal microorganism’s unique amplicon sequence variants (ASVs) abundance on sea buckthorn. The color intensity is proportional to the relative abundance of fungal microorganism ASVs.

A heatmap diagram illustrates the distribution of the most abundant fungal ASVs (Figure 4). Hierarchical cluster analysis showed that the microbial community structure displays different patterns between UB and RB groups, while samples collected within the same ripening stage feature similar community structure. The core microbiomes detected in both samples comprised of ASV1 and ASV3 assigned to *Aureobasidium pullulans* (Figure 4; Table S2). At the beginning of ripening, besides the *Aureobasidium pullulans* the most abundant ASVs were ASV4 (7.6%) and ASV14 (2.4%) (both *Taphrina carpini*), ASV10 (4.7%) (Taphrina), ASV6 (6.8%) (*Cladosporium*), and ASV20 (1.6%) (*Filobasidium wieringae*). During the ripening of the berries, their prevalence diminished to less than 1%. A dramatic change was observed between the beginning and the end of the ripening period, mainly due to the increase of fungal microorganisms belonging to *Metschnikowia spp*. (ASVs 2, 5, 7, 9, 11, 22, and 30). The overall abundance of ASVs assigned to *Metschnikowia* in RB berries reached 44%, while on the unripe berries they consisted only 0.7%. 

### 3.3. Sea-Buckthorn-Associated Cultivable Potential Biocontrol Yeasts and Their Produced Volatiles

By applying cultivation techniques, more than 20 yeast strains were isolated from the surface of sea buckthorn berries. Yeasts were subjected for primary species identification by morphological colony analysis and bright field microscopy with following fingerprinting of PCR-amplified sequences of internal transcribed spacers 1 and 2 including 5.8S ribosomal RNA gene. For further analysis of VOC production, selected strains proceeded sequencing-based species level confirmation and deposition of sequences in NCBI. 

**Figure 5:**
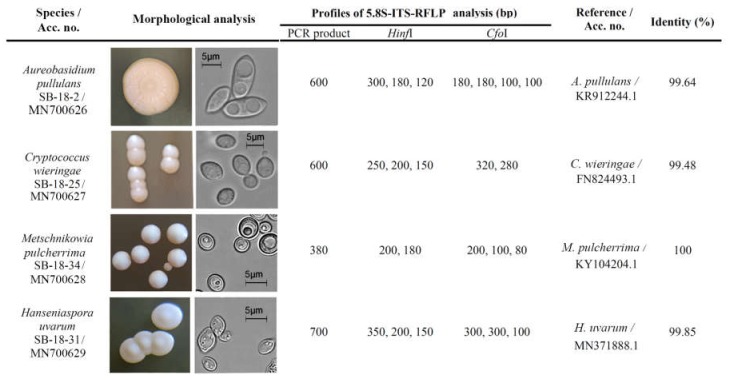
Identification of yeasts isolated from sea buckthorn surface.

Four yeast strains, belonging to different genera identified by metagenomics analysis of UB and RB microbiota with referred potential biocontrol properties [15,16,40] (Table S1, Figure 5), were selected for VOCs analysis. Morphologically different SB-18-2 and SB-18-25 yeast strains were recovered from UB berries while SB-18-34 and SB-18-31 yeast strains were isolated from RB berries. Basing on the electrophoretic analysis, similar size (about 600 bp) ITS regions were PCR-amplified from SB-18-2 and SB-18-25 strains, and differed in *HinfI* and *CfoI* restriction patterns (Figure 5). The sequence identity match of SB-18-2 yeast was 99.64%, comparing to that of *Aureobasidium pullulans* culture YY11 deposited in GenBank. SB-18-25 yeast strain was assigned to *Cryptococcus wieringae* (syn. *Filobasidium wieringae*) basing on RFLP profile and 99.48% similarity to *C. wieringae* CBS:11709 culture. Following the genomic DNA purification, 380 bp and 700 bp PCR products were amplified from SB-18-34 and SB-18-31 yeast strains, respectively. Their PCR-RFLP profiles corresponded to *Metschnikowia* and *Hanseniaspora* yeast species [41]. According to the high similarity to sequences depositedin GenBank, SB-18-34 strain was identified as *Metschnikowia pulcherrima* and SB-18-31 as *Hanseniaspora uvarum* yeast species (Figure 5).

*Aureobasidium pullulans* SB-18-2 belongs to genus, which is the most abundant in UB samples (31.4%), and of significant representation (40.6%) in RB samples. *Metschnikowia pulcherrima* SB-18-34 represents genus, which is the most abundant (44.5%) in RB samples. *Cryptococcus wieringae* SB-18-25 belongs to genus, more prevalent (1.9%) on the surface of UB berries than on the RB berries (0.2%). *Hanseniaspora uvarum* SB-18-31 is a member of genus, which is the least dominant among the selected genera with only 0.5% and 0.2% occurrence in UB and RB samples, respectively.

The gas chromatographic-mass spectrometric analysis revealed 29 compounds that were exclusively present in the headspace of four yeast species and occurred significantly more abundant compared to those of control samples. The yeast released volatiles were composed of 11 esters, 6 alcohols, 5 volatile fatty acids, 3 ketones, 2 aromatics, and 2 compounds bearing both ester and aromatic moieties (Table 2).

**Figure 6:**
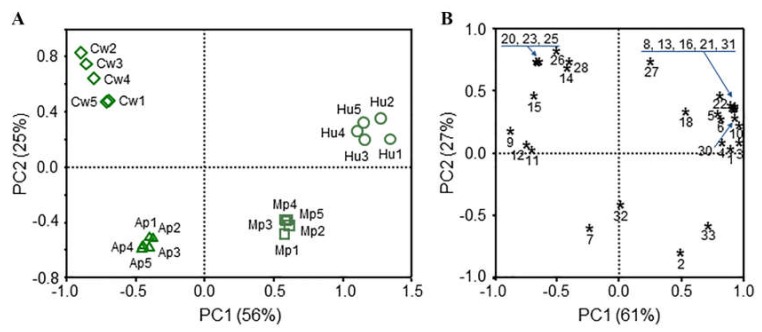
Associations between blends and their components of four species of yeasts populating sea buckthorn berries. Associations are visualized by principal component analysis (PCA). (**A**) Distribution of odor blends released by *C. wieringae* (Cw, diamond shape), *A. pullulans* (Ap, triangle shape), *M. pulcherrima* (Mp, square shape), and *H. uvarum* (Hu, circle shape). n=5 for each species. (**B**) Distribution of volatile compounds composing the blends. Stars represent the volatile compounds indicated by numbers. Names of the compounds are listed in Table 2.

The principal component analysis revealed that the volatile blend of any of the four yeast species was clearly separated from each other. The first principal component axis accounted for 56% of the total variation and to a large extent explained separation between volatile blends of *C. wieringae* versus *H. uvarum* yeasts and *A. pullulans* versus *M. pulcherrima* yeasts (Figure 6A). Volatile profiles of *C. wieringae* yeasts were characterized by five alcohols (9, 11, 14, 15, and 23), three volatile fatty acids (25, 26, and 28), and two ketones (12 and 20) which were exclusively present or occurred in the large amounts compared to those released by *H. uvarum* yeasts (Figure 6B, Table 2). Ten esters were unique for the volatile blends of *H. uvarum* yeasts in addition to acetic acid (22), 2-hydroxy-3-butanone (18), and 2-phenylethanol (33) with amounts larger compared to those of *C. wieringae* yeasts (Figure 6B, Table 2). *M. pulcherrima* yeasts released the larger amounts of esters (1, 3, 4, and 10), while *A. pullulans* yeasts released the higher amounts of two alcohols (ethanol (2), butanol (11)) (Table 2).

The second PC axis accounted for 25% of the total variation and explained the separation between volatile blends of *A. pullulans* and *C. wieringae* species and in part between volatile blends of *M. pulcherrima* and *H. uvarum* yeasts (Figure 6A). 6-Methyl 5-hepten-2-one (20), 6-methyl-5-hepten-2-ol (23), propionic acid (25) and 2-methylpropionic acid (26) were unique volatiles released by *C. wieringae,* while only ethyl 3-methylbutanoate (7) was specific for *A. pullulans* (Figure 6B, Table 2). Ethyl butanoate (5), ethyl 2-methylbutanoate (6), 3-methylbutyl propionate (13), ethyl hexanoate (16), ethyl octanoate (21), and 2-phenylethyl propionate (31) were exclusively present in volatile blends of *H. uvarum* yeasts, and phenylmethanol (32) was unique for *M. pulcherrima* (Figure 6B, Table 2). 

## 4. Discussion

The current study, for the first time, uncovered the structure of fungal microorganism communities colonizing the surface of sea buckthorn berries at different ripening stages in natural habitat. For a comprehensive analysis, we have applied next generation sequencing and observed the wide diversity of eukaryotic microorganisms on tested berries. The total abundance of fungal microorganisms changed significantly during the period of fruit ripening. The microbial alpha-diversity—estimated by the Simpson’s index, Shannon’s diversity index, and Pielou’s evenness index—was highest among the fungal community on unripe berries. The analysis of the fungal microbiota structure revealed clear differentiation between unripe and mature berries, as demonstrated by the Bray–Curtis beta-diversity analysis. According to our data, unripe sea buckthorn berries were dominated by *Aureobasidium*, *Taphrina*, *Cladosporium*, and *Filobasidium* (syn. *Cryptococcus*) genera microorganisms, while on ripe berries prevailed *Aureobasidium* and *Metschnikowia* (Table S1, Figure 2). It was previously demonstrated that *Cryptococcus*, *Rhodotorula*, *Sporobolomyces*, and *Aureobasidium pullulans* yeasts were associated with the early stage of maturation of fruits and berries, such as apples, grapes, nectarines, etc. [29,32,42]. In our previous work, it was demonstrated that Dothioraceae family fungal microorganisms prevailed on sea buckthorn berries, which at the lower taxonomic level were attributed to uncultured *Aureobasidium* [33]. Our results were partially consistent with previous findings, since most fungal microorganisms present on sea buckthorn were represented by *Aureobasidium*. However, the overall structure of fungal microbiota varies most likely depending on the differences in climatic conditions, berry harvesting period (previously—September 2016; in this study—July 2018), localization of sea buckthorn plantation, etc. 

Various microorganisms act as biological control agents of phytopathogens and have a role in postharvest decay and toxin contamination by emitting volatile organic compounds [40,43]. Several biocontrol yeasts (e.g., *Candida oleophila, Aureobasidium pullulans, Metschnikowia fructicola, Cryptococcus albidus, Saccharomyces cerevisiae*) have already been used for the production of commercial plant protection products, encouraging the deeper exploration of the fascinating traits and application possibilities of other yeasts as well [16,44,45]. Even the different strains of the same yeast species are able to produce highly different amounts of the same VOCs (e.g., *A. pullulans* [43]), thus it is important to investigate volatiles of yeasts strains within the species that are already proven to possess biocontrol traits (like *Aureobasidium pullulans* [43,46], *Metschnikowia pulcherrima* [46], and *Hanseniaspora uvarum* [47]), or are less investigated, but closely related to bioactive yeasts (e.g., potential biocontrol yeast *Cryptococcus wieringae* which belongs to the same genus as *C. albidus, C. laurentii*, and C. flavus [16,48,49,50]).

*Aureobasidium pullulans* is well-known yeast-like fungus hosting on plants, mostly present in a cooler climate like that of central Europe and less related to grapes grown in warm areas [51]. It was revealed that some *Aureobasidium* species demonstrated biological control activity against phytopathogens, such as *Botrytis*, *Bacillus, Colletotrichum, Penicillium* [43,52,53]. The antagonistic activity of *A. pullulans* mainly includes nutrient competition and production of volatile organic compounds or glucanases, chitinases, and extracellular proteases [40,46]. *Taphrina* and *Cladosporium* were the next numerous fungal microorganisms observed on unripe sea buckthorn berries in our study (Table S1, Figure 2). *Taphrina* genus fungi are known as biotrophic plant pathogens that cause plant and fruit disease symptoms, which may be ascribed to the ability to synthesize and modulate different plant hormones [54]. *Cladosporium* is considered as ubiquitous fungi, with some species providing numerous antifungal agents [55], while others causing plant or human diseases [56,57]. *Cryptococcus* encloses species producing biocontrol agents against many pathogens [16,50], while certain species can cause infections in humans and animals [58]. 

During the ripening of different fruits and berries, the oxidative or weakly fermentative yeast populations—such as *Candida*, *Hanseniaspora*, *Metschnikowia*, and *Pichia*—increased in frequency [42]. *Hanseniaspora* spp. have been frequently found on the surface of different fruits, e.g. apples, grapes, or strawberries [59,60]. Numerous yeasts belonging to the *Hanseniaspora* genus display antagonistic properties against the development of fruit spoilage causing molds [40,61]. *Metschnikowia* spp. isolated from fruit are sugar-loving yeasts and their populations are most likely stimulated by juices diffusing from the damaged surface of mature fruit [42]. This yeast genus includes several biocontrol agents, which are very effective against fruit spoilage fungi [62]. The biocontrol abilities of *M. pulcherrima* have been mainly assigned either to competition for nutrients or to the production of volatile organic compounds [15,46]. The competition of *M. pulcherrima* for iron was reported to play a substantial role in biocontrol interactions [63]. *Metschnikowia* spp. are linked to several groups of nectarivorous insects, suggesting that this specificity could be correlated to the services provided by the yeast to the insect or vice versa [64,65]. 

Yeasts produce chemo-diverse blends of volatiles comprised of aldehydes, ketones, alcohols, volatile fatty acids, esters, aromatics, heterocyclic compounds, hydrocarbons, terpenoids, and some others [66,67]. Ethanol is probably the most known volatile metabolite of yeasts derived from carbohydrate catabolism and fermentation [66]; it is one of the major volatiles collected from the headspace of *H. uvarum*, *M. pulcherrima*, and *C. wieringae* yeasts. Amounts of ethyl acetate, another metabolite of carbohydrate catabolism, prevailed in the VOCs produced exclusively by *H. uvarum and M. pulcherrima* yeasts. It is a valuable solvent for extracting compounds with antimicrobial properties [68] and possess minor inhibitory effect for *Botrytis cinerea* [69]. In addition to ethyl acetate, 10 esters were unique for the volatile blends of *H. uvarum* yeasts; these compounds originated from amino acid synthesis and degradation pathways [66]. Prevalence of esters in volatile blends of *H. uvarum* has been reported in previous studies [70,71]; however, ethanol being the third largest component of the blend in our experiments, has not been determined by Babcock et al. [71] or Piper et al. [70]. High production of ethanol, 3-methylbutanol, 2-phenylethanol, ethyl acetate, and 3-methylbutyl acetate were reported for *M. pulcherrima* and *A. pullulans* yeasts [15]. The data published are comparable with the results of our study in terms of large production of alcohols by both yeast species; however, we were not able to detect ethyl acetate in the headspace of *A. pullulans*. To date, there is no report on VOCs of *C. wieringae* yeast. Volatile organic compounds produced by *A. pullulans* that were found in our study (namely 2-phenylethanol, 2-methylpropanol, 2-methylbutanol, and 3-methylbutanol) had been previously shown to control postharvest fruit pathogens (*B. cinerea, Colletotrichum acutatum, Penicillium expansum, Penicillium digitatum*, and *Penicillium italicum*) [43]. All these alcohols were produced by *A. pullulans* SB-18-2, *M. pulcherrima* SB-18-34, *C. wieringae* SB-18-25, and *H. uvarum* SB-18-31 but in different amounts, suggesting the potential biocontrol capabilities of these strains. 2-Phenylethanol is one of the main VOCs produced by various yeast species (e.g. *Cyberlindnera jadinii, Lachancea thermotolerans, Candida intermedia, Candida friedrichii,* *Saccharomyces cerevisiae, Wickerhamomyces anomalus)* affecting numerous pathogens (e.g. Aspergillus carbonarius, Aspergillus flavus, Aspergillus ochraceus, Penicillium digitatum, Phyllosticta citricarpa) (see [40] and references therein). Among tested strains, *H. uvarum* SB-18-31 and *M. pulcherrima* SB-18-34 produced the highest amount of 2-phenylethanol, whereas *A. pullulans* SB-18-2 excelled at ethanol generation, while volatilome of *C. wieringae* SB-18-25 was dominated by 3-methylbutanol. Ethyl acetate was the dominating volatile of *H. uvarum* SB-18-31 and *M. pulcherrima* SB-18-34. 2-Phenylethyl acetate was produced in significant amounts (comparing to other tested yeast strains) by *H. uvarum* SB-18-31 and had been previously reported to contribute to growth inhibition of *A. ochraceus* growth and ochratoxin A production during processing of coffee [47]. 

Due to the extremely sensitive olfaction system, insects are capable to use microbial volatiles to search for suitable feeding and oviposition sites [18,72]. Emissions from microorganisms associated with different development stages of fruits and berries contribute to the information about habitat suitability [73] and it was demonstrated that insects recognized yeast communities based on their specific volatile profiles [74,75,76]. Most of the volatile compounds (2-phenyl ethanol, 2-methylbutanol, 3-methylbutanol, ethyl acetate, 2-phenylethyl acetate, ethyl propionate, and others) produced by yeast in our study had been suggested to play a role in modulating behavior of *Rhagoletis batava* flies [67]. Examples of microbial VOC application for pest control already demonstrated their high potential for use in integrated pest management programs [77]. Identification of attractive and repellent odors from the yeasts associated with berries at preferred and unsuitable stages for pest insect feeding and oviposition would provide a base for the development of environment-friendly pull-push pest control for *Rhagoletis batava* flies, a major pest of the sea buckthorn.

## 5. Conclusions

The metagenomic approach based on the sequencing of ITS2 region of fungal rDNA revealed changes in the fungal community associated with the ripening of sea buckthorn berries. Investigation of alpha and beta diversity of the fungal community, analysis of representative microbial ASVs showed a clear separation among inhabitants of unripe (UB) and ripe berries (RB). Based on results of metagenomics analysis, unripe berry samples prevalently had *Aureobasidium*, *Taphrina*, and *Cladosporium* genera yeasts; while ripe berries were dominated by *Aureobasidium* and *Metschnikowia*. Potential biocontrol yeasts *Aureobasidium pullulans* and *Cryptococcus wieringae* were isolated from sea buckthorn surface at the early harvesting stage, while *Metschnikowia pulcherrima* and *Hanseniaspora uvarum* were amplified from ripe berries and applied for the analysis of produced volatile organic compounds. Eleven esters, six alcohols, five volatile fatty acids, three ketones, two aromatics, and two compounds bearing both ester and aromatic moieties were isolated from the headspaces of four yeast species and identified by gas chromatography and mass spectrometry techniques. Principal component analysis revealed that volatile blends of all four yeast species were clearly separated from each other. 

## Figures and Tables

**Table 1:** Total sequences obtained for eukaryotic microbial community for unripe (UB) and ripe (RB) sea buckthorn samples.

	Reads Obtained	High Quality Reads	ASV	Shanon Diversity	Pielou Eveness	Simpson Index
**UB1**	103,024	49,891	300	5.26	0.64	0.94
**UB2**	164,922	110,782	419	5.00	0.58	0.91
**UB3**	89,720	43,458	295	5.37	0.66	0.94
**RB1**	114,814	76,641	190	3.71	0.49	0.86
**RB2**	135,098	92,822	240	3.795	0.48	0.85
**RB3**	119,056	80,944	171	3.21	0.44	0.79
**Total**	726,634	454,538	1615			

**Table 2:** Odor blends of four yeast species and control sampled by SPME technique.

No.	Compound	CAS No.	RI	GR	*H. uvarum*	*M. pulcherrima*	*A. pullulans*	*C. wieringae*	Control
1	Ethyl acetate	141-78-6	898	ES	26.26 ± 4.63	8.98 ± 0.28	0	0	0.06 ± 0.02
2	Ethanol	64-17-5	902	OH	4.58 ± 0.36	4.89 ± 0.42	6.26 ± 0.82	0.39 ± 0.03	0.10 ± 0.02
3	Ethyl propionate	1105-37-3	915	ES	1.00 ± 0.05	0.24 ± 0.01	0	0	0.01 ± 0.001
4	2-Methylprop-1-yl acetate	110-19-0	985	ES	0.31 ± 0.09	0.07 ± 0.01	0	0	0
5	Ethyl butanoate	105-54-4	1013	ES	0.05 ± 0.03	0	0	0	0
6	Ethyl 2-methylbutanoate	7452-79-1	1033	ES	0.02 ± 0.01	0	0	0	0
7	Ethyl 3-methylbutanoate	108-64-5	1050	ES	0	0	0.04 ± 0.01	0	0
8	Butyl acetate	123-86-4	1063	ES	0.28 ± 0.03	0	0	0	0.10 ± 0.02
9	2-Methylpropanol	78-83-1	1095	OH	0.42 ± 0.07	0.98 ± 0.08	1.04 ± 0.13	1.70 ± 0.11	0
10	3-Methylbutyl acetate	123-92-2	1105	ES	13.28 ± 0.5	0.40 ± 0.02	0.15 ± 0.05ns	0.07 ± 0.01ns	0.10 ± 0.07
11	Butanol	71-36-3	1149	OH	0	0	0.13 ± 0.03	0.09 ± 0.01	0
12	2-Heptanone	110-43-0	1170	KT	0	0	0.07 ± 0.01	0.06 ± 0.01	0
13	3-Methylbutyl propionate	105-68-0	1176	ES	0.62 ± 0.09	0	0	0	0
14	2-Methylbutanol	137-32-6	1207	OH	1.63 ± 0.28	1.77 ± 0.24	0.84 ± 0.18	6.47 ± 1.21	0
15	3-Methylbutanol	123-51-3	1213	OH	3.28 ± 0.37	5.95 ± 0.56	4.07 ± 0.51	10.39 ± 0.36	0.21 ± 0.11
16	Ethyl hexanoate	123-66-0	1224	ES	0.02 ± 0.01	0	0	0	0
17	Styrene*	100-42-5	1238	AR	0.20 ± 0.04ns	0.13 ± 0.03ns	0.07 ± 0.01ns	0.16 ± 0.01ns	0.14 ± 0.06ns
18	2-hydroxy-3-butanone	513-86-0	1273	KT	0.15±0.04	0.14 ± 0.01	0	0.06 ± 0.01	0
19	2,5- Dimethyl pyrazine*	123-32-0	1316	PY	0.02±0.01ns	0.01 ± 0.001ns	0.03 ± 0.01ns	0.04 ± 0.01ns	0.03 ± 0.02ns
20	6-Methyl 5-hepten-2-one	110-93-0	1327	KT	0	0	0	0.93 ± 0.10	0
21	Ethyl octanoate	106-32-1	1430	ES	0.12 ± 0.01	0	0	0	0
22	Acetic acid	64-19-7	1449	FA	1.15 ±0.25	0	0.01 ± 0.008	0.07 ± 0.02	0
23	6-Methyl-5-hepten-2-ol	1569-60-4	1461	OH	0	0	0	0.08 ± 0.02	0
24	2-Ethylhexanol*	104-76-7	1488	OH	0.02 ± 0.001ns	0.02±0.01ns	0.02±0.001ns	0.02±0.004ns	0.03±0.01ns
25	Propionic acid	1979-09-04	1528	FA	0	0	0	0.03 ± 0.004	0
26	2-Methylpropionic acid	79-31-2	1562	FA	0	0	0	0.18 ± 0.04	0
27	Butanoic acid	107-92-6	1634	FA	0.03 ± 0.01	0	0	0.03 ± 0.01	0
28	3-Methylbutanoic acid	503-74-2	1703	FA	0.07 ± 0.02	0.01 ± 0.001	0.06 ± 0.04	0.71 ± 0.25	0
29	Methoxy-phenyl-oxime*		1767	IM	0.03 ± 0.01ns	0.01 ± 0.003	0.01 ± 0.004	0.03 ± 0.01ns	0.03 ± 0.01ns
30	2-Phenylethyl acetate	103-45-7	1795	ES/AR	3.52 ± 0.64	0.04 ± 0.01	0	0	0
31	2-Phenylethyl propionate	122-70-3	1858	ES/AR	0.11 ± 0.02	0	0	0	0
32	Phenylmethanol	1960-12-08	1856	AR	0	0.03 ± 0.01	0	0	0
33	2-Phenylethanol	1960-12-08	1894	AR	3.60 ± 0.26	3.45 ± 0.65	2.28 ± 0.45	0.21 ± 0.03	0.03 ± 0.01

Compounds indicated by * mark were excluded from PCA; CAS No—chemical abstract service number; RI—retention index (DB-Wax fused silica capillary column 30 m x 0.25 mm i.d., 0.25 µm film thickness); GR—group of chemical compound; ES—ester; OH—alcohol; KT—ketone; AR—aromatic; PY—pyrazine; FA—fatty acid; IM—imine; all values in the columns headed by yeast names are the absolute amounts expressed as areas under the chromatographic peaks and have to be read as numbers times 100000 ± standard error of the mean followed by the “ns” are not significantly different compare to control (nonparametric Mann–Whitney U test, *P* < 0.05); control samples were obtained by collecting background volatiles from YPD-agar plates without yeast.

## Supplementary Materials

The following are available online at https://www.mdpi.com/2076-2607/8/3/456/s1, Figure S1: Rarefaction curves at a genetic distance of 3% for each sample, Table S1: Fungal taxonomy abundance count for sea buckthorn samples at different ripening stages from phylum to species level, Table S2: Relative abundance of the most common ASVs.
