# Correction: Lukša, J., et al. Fungal Microbiota of Sea Buckthorn Berries at Two Ripening Stages and Volatile Profiling of Potential Biocontrol Yeasts. *Microorganisms* 2020, *8*, 456

**DOI:** 10.3390/microorganisms8071090

**Published:** 2020-07-21

**Authors:** Juliana Lukša, Iglė Vepštaitė-Monstavičė, Violeta Apšegaitė, Laima Blažytė-Čereškienė, Ramunė Stanevičienė, Živilė Strazdaitė-Žielienė, Bazilė Ravoitytė, Dominykas Aleknavičius, Vincas Būda, Raimondas Mozūraitis, Elena Servienė

**Affiliations:** 1 Laboratory of Genetics, Institute of Botany, Nature Research Centre, Akademijos str. 2, Vilnius LT-08412, Lithuania; juliana.luksa@gamtc.lt (J.L.); igle.vepstaite@yahoo.com (I.V.-M.); ramune.staneviciene@gamtc.lt (R.S.); zivile.strazdaite-zieliene@gamtc.lt (Ž.S.-Ž.); bazile.ravoityte@gamtc.lt (B.R.); 2 Laboratory of Chemical and Behavioral Ecology, Institute of Ecology, Nature Research Centre, Akademijos str. 2, Vilnius LT-08412, Lithuania; violeta.apsegaite@gamtc.lt (V.A.); laima.blazyte@gamtc.lt (L.B.-Č.); dominykas.aleknavicius@gamtc.lt (D.A.); vincas.buda@gamtc.lt (V.B.); raimondas.mozuraitis@gamtc.lt (R.M.)

The authors wish to make the following corrections to this paper [1]:

The authors in the Funding section of [1] found an error. Consequently, the authors wish to correct “This research was funded by European Social Fund/European Regional Development Fund under grant agreement with the Research Council of Lithuania (LMTLT), grant number 09.3.3-LMT-K-712-01-0099.” to “This project has received funding from European Social Fund (project No 09.3.3-LMT-K-712-01-0099) under grant agreement with the Research Council of Lithuania (LMTLT).”

The authors would like to apologize for any inconvenience caused to the readers by these changes.
