# Supplementary figures and images for: Fungal Microbiota of Sea Buckthorn Berries at Two Ripening Stages and Volatile Profiling of Potential Biocontrol Yeasts
Source: Microorganisms. 2020 Mar 23;8(3):456. doi: 10.3390/microorganisms8030456 (PMC7143951; doi:10.3390/microorganisms8030456)

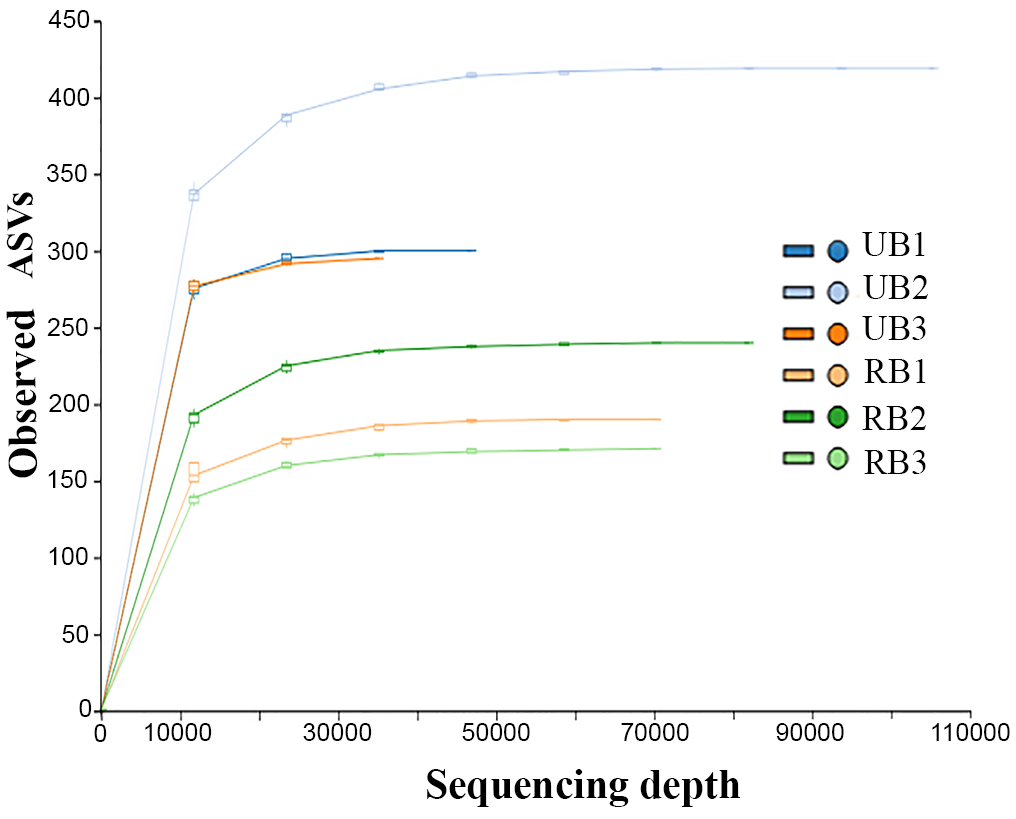

Supplement: Supplementary file 1 [file microorganisms-08-00456-s001.zip › Figure S1.tif]
